# Human papilloma virus and breast cancer

**DOI:** 10.1038/sj.bjc.6602944

**Published:** 2006-01-10

**Authors:** K Altundag, M Z Baptista

**Affiliations:** 1 Department of Medical Oncology, Hacettepe University Institute of Oncology, Ankara, Turkey; 2 Department of Clinical Oncology, Hospital Maternidade De Campinas, Campinas, SP, Brazil

**Sir**,

We read with great interest the article by Kan *et al* (2005). In their study they confirmed presence of human papilloma virus (HPV) 18 gene sequences in breast tumour. Our concern is that this study could not define whether HPV 18 is located in breast cancer cells or other cells such as stromal cells present at microenvironment of breast tumour. If it is shown that HPV 18 gene sequences are located inside breast cancer cell, this information may further strengthen close association between carcinogenesis and HPV 18. Our another concern is transmission of HPV 18 to the breast. One study showed that HPVs are present in cancers occurring in human nipple milk ducts and that these cancers have the typical histological features of HPV-induced human cancers (De Villiers *et al*, 2005). Therefore, we can speculate that HPV might be associated more with ductal histology than with lobular histology. Therefore, correlation between breast cancer histology type and presence of HPV 18 genome sequence may give us some clue to understand pathogenesis of HPV-induced carcinogenesis.
